# Supplementary material for: Exploring the Role of PI3P in Platelets: Insights from a Novel External PI3P Pool
Source: Biomolecules. 2023 Mar 24;13(4):583. doi: 10.3390/biom13040583 (PMC10135486; doi:10.3390/biom13040583)
Supplement: Supplementary file 1 [file biomolecules-13-00583-s001.zip › biomolecules-2251437-supplementary-correction.pdf]

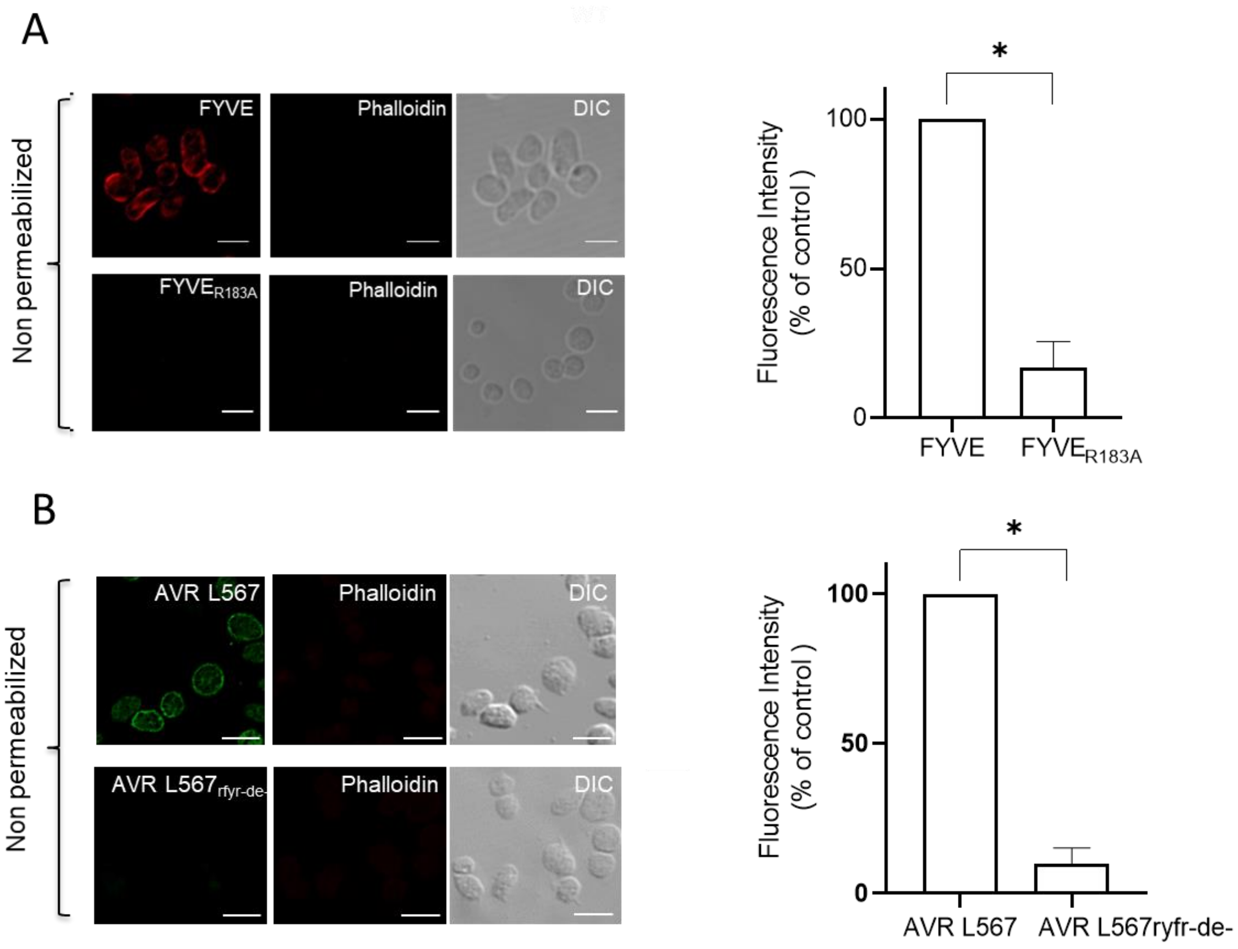

Supplemental Figure S1 : Detection of an external PI3P pool in mouse platelets. Fixed, washed resting mouse platelets were incubated with the FYVE (A) and the AVR L567 (B) PI3P probes or their inactive mutants for 60 minutes. The F-actin probe, ^{488}Alexa-conjugated phalloidin, was used to monitor platelet membrane integrity. After washing, platelets were fixed and analyzed using confocal microscopy. Differential interference contrast (DIC) was used to identify platelets in the field. Images shown are representative of 3 to 5 independent experiments. Scale bar: 5 µm. The fluorescence intensity was quantified, and the results are expressed as a percentage of control (fluorescence of the FYVE or AVR L567 probes). Wilcoxon test compared the different conditions to control, *p ≤0.05.

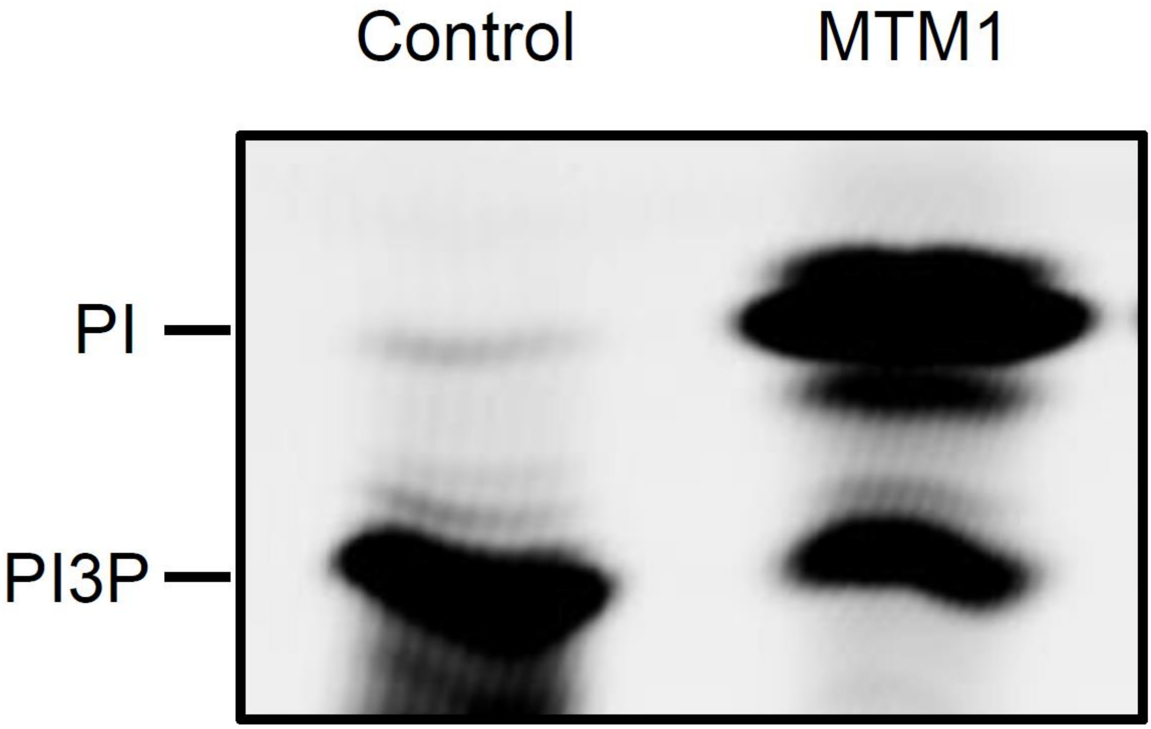

Supplemental Figure S2: Control of MTM1 enzymatic activity. To control the enzymatic activity of recombinant MTM1, we used di-C16-^{bodipy}PI3P as a substrate. After incubating for 30 minutes at 30°C, we extracted and separated lipids by thin-layer chromatography. Fluorescent lipids were visualized using a Typhoon image scanner equipped with a blue laser (488 nm). The position of C16-PI3P and C16-PI on the thin-layer chromatography is indicated in the figure. A control lane corresponding to di-C16-^{bodipy}PI3P incubated for 30 minutes at 30°C in the absence of recombinant MTM1 is included. The results shown are representative of 3 independent experiments.

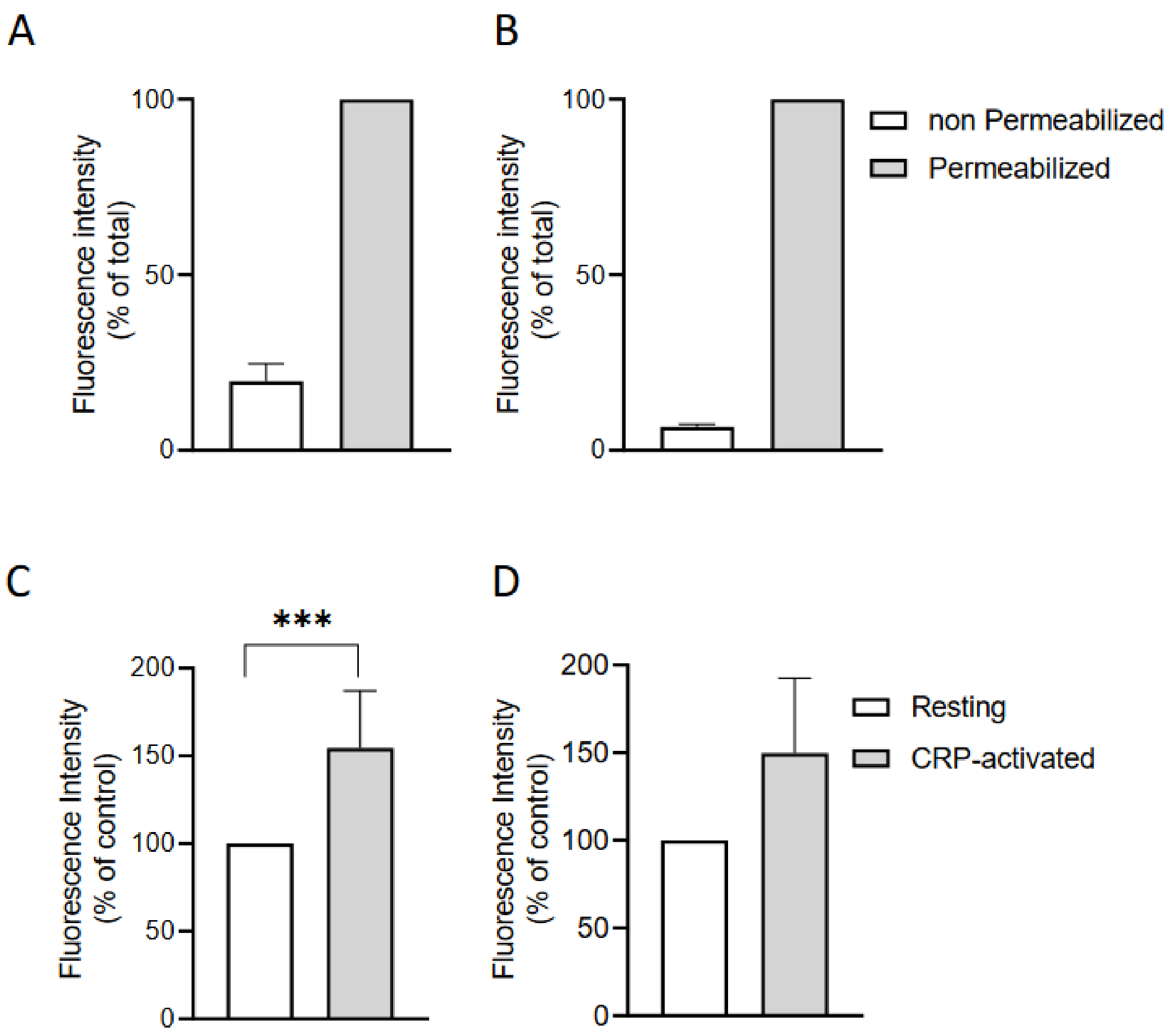

Supplemental Figure S3: Estimation of the percentage of PI3P in the external pool and effect of platelet stimulation. PI3P labeling with the FYVE probe was quantified by flow cytometry in fixed, non-permeabilized (external PI3P) and permeabilized (total PI3P), resting human (A) or mouse (B) platelets. PI3P labeling with the FYVE probe was quantified by flow cytometry in fixed, non-permeabilized human (C) or mouse (D) platelets stimulated or not by collagen-related peptide (CRP, 1 μg/ml for 10 minutes in the presence of integrilin, an αIIbβ3 inhibitor to prevent aggregation). Results are expressed as mean ± SEM of 8 and 3 independent experiments for human and mouse platelets, respectively. In C and D Wilcoxon test compared CRP-activated conditions to resting, ***p ≤0.001.

Supplementary Table S1. List of primers used in this study.

Plasmid constructions	Backbone plasmid & Enzymes used	Insert	Primers sequences
pmCherry-C1-FYVE (HRS)	pmCherry-C1<br>Xho1/<br>BamH1	FYVE (HRS) amplified from pGEX-2TK-FYVE (HRS)	Forward :<br>CCGCTCGAGCAGAGAGAGCCCCAGACT<br>Reverse :<br>CGCGGATCCTACCTGTTCAGCTGCTCGT
pGEX-4T-1-mCherry-FYVE (HRS)	pGEX-4T-1<br>BamH1/<br>Sal1	mCherry-FYVE (HRS) amplified from pmCherry-C1-FYVE (HRS)	Forward :<br>CCGGGATCCATGGTGAGCAAGGGCGAGGAG<br>Reverse :<br>CGCGTCGACCTACCTGTTCAGCTGCTCGT
pmCherry-C1-PH (GRP1)	pmCherry-C1<br>Xho1/<br>BamH1	PH (GRP1) amplified from pGEX-2TK- PH (GRP1)	Forward :<br>CCGCTCGAGGACCGTTTAAGATCCCAGAAGA<br>Reverse :<br>CGCGGATCCTATTTCTTATTGGCAATCCTCCT
pGEX-4T-1-mCherry- PH (GRP1)	pGEX-4T-1<br>BamH1/<br>Sal1	mCherry- PH (GRP1) amplified from pmCherry-C1-PH (GRP1)	Forward :<br>CCGGGATCCATGGTGAGCAAGGGCGAGGAG<br>Reverse :<br>CGCGTCGACCTATTTCTTATTGGCAATCCTCCT
pmCherry-C1-PH (FAPP1)	pmCherry-C1<br>Xho1/<br>BamH1	PH (FAPP1) amplified from pGEX-2TK-PH (FAPP1)	Forward :<br>CCGCTCGAGCAATGGAGGGGGTGTTGTACAA<br>Reverse :<br>CGCGGATCCTACCTTGTATCAGTCAAACATG
pGEX-4T-1-mCherry-PH (FAPP1)	pGEX-4T-1<br>BamH1/<br>Sal1	mCherry-PH (FAPP1) amplified from pmCherry-C1-PH (FAPP1)	Forward :<br>CCGGGATCCATGGTGAGCAAGGGCGAGGAG<br>Reverse :<br>CGCGTCGACCTACCTTGTATCAGTCAAACATG
pmCherry-C1-PH (PLC-δ1)	pmCherry-C1<br>Xho1/<br>BamH1	PH (PLC-δ1) Amplified from pGEX-2TK-PH (PLC-δ1)	Forward :<br>CCGCTCGAGGACACGGGCTCCAGGATGA<br>Reverse :<br>CGCGGATCCTACTTCTGCCGCTGGTCCATG
pGEX-4T-1-mCherry- PH (PLC-δ1)	pGEX-4T-1<br>BamH1/<br>Sal1	mCherry- PH (PLC-δ1) amplified from pmCherry-C1-PH (PLC-δ1)	Forward :<br>CCGGGATCCATGGTGAGCAAGGGCGAGGAG<br>Reverse :<br>CGCGTCGACCTACTTCTGCCGCTGGTCCATG
pmCherry-C1-PHD2x (ING2)	pmCherry-C1<br>Xho1/<br>BamH1	PHD2x (ING2) Amplified from pEGFP-PHD2x (ING2)	Forward :<br>CCGCTCGAGAATCCGCGTCACCTGTTGAGT<br>Reverse :<br>CGCGGATCCTACCTCGATCTTCTATCCTTTT
pGEX-4T-1-mCherry-PHD2x (ING2)	pGEX-4T-1<br>BamH1/<br>Sal1	mCherry- PHD2x (ING2) amplified from pmCherry-C1- PHD2x (ING2)	Forward :<br>CCGGGATCCATGGTGAGCAAGGGCGAGGAG<br>Reverse :<br>CGCGTCGACCTACCTCGATCTTCTATCCTTTT
